# Efficacy and safety of acupuncture combined with Western medicine in the treatment of ankylosing spondylitis: A systematic review and meta-analysis

**DOI:** 10.1097/MD.0000000000042468

**Published:** 2025-05-23

**Authors:** Xindan Cao, Yadan Zhang, Zhihui Xiao, Jianhong Peng

**Affiliations:** a Institute of Graduate Studies, Guangzhou University of Chinese Medicine, Guangzhou, China; b Department of Rheumatology, Dongguan Hospital of Traditional Chinese Medicine, Dongguan, China.

**Keywords:** acupuncture, ankylosing spondylitis, meta-analysis, randomized controlled trial

## Abstract

**Background:**

An autoimmune disease called ankylosing spondylitis (AS) is known to cause stiffness and pain in the joints. Acupuncture is a traditional Chinese medicine that has been used extensively to treat AS. It has several advantages, including safety, affordability, and few adverse effects. However, there is limited data to support its therapeutic effects. As a result, the goal of the current study is to assess acupuncture’s reliability in treating AS in its entirety.

**Methods:**

Seven databases were searched from January 1, 2000, to July 31, 2024, for randomized controlled trials (RCTs) on acupuncture in conjunction with Western medicine to treat AS. The retrieved data was meta-analyzed using Review Manager 5.4 and Stata 17.0. The updated Cochrane risk of bias tool was used to evaluate the bias risk in RCTs.

**Results:**

The results revealed that combining Acupuncture and Western medicine led to better outcomes as far as effective rate (RR = 1.25, 95% confidence intervals (CI): 1.16–1.34); thoracic mobility (MD = 0.58, 95% CI: 0.43–0.73); Schober test (SMD = 0.83, 95% CI: 0.57–1.09); bath ankylosing spondylitis disease activity index (MD = –1.11, 95% CI: −1.46 to −0.76); visual analog scale for pain (MD = –1.02, 95% CI: −1.44 to −0.60); CRP (MD = –2.79, 95% CI: −4.14 to −1.43); ESR: (MD = –5.33, 95% CI: −6.63 to −4.02); and adverse reactions (RR = 0.58, 95% CI: 0.35–0.95) in contrast to treating AS with just Western treatment.

**Conclusion:**

When paired with Western therapy, acupuncture improves the effective rate, functional scores, and symptoms of people with AS while lowering adverse reactions.

## 1. Introduction

Ankylosing spondylitis (AS) is a chronic inflammatory illness that mostly affects the axial joints, including the sacroiliac joints and spine.^[1]^ Clinically, its early symptoms include low back pain and morning stiffness, and in later stages, due to spinal ankylosis, it may lead to complications such as limited lumbar spine mobility, reduced thoracic spine motion, and severe osteoporosis, which ultimately cause a decrease in the quality of life or even disability of the patients, and some serious ones may have spinal deformities.^[1]^ The prevalence of AS is estimated to range from 0.03% to 1.8% in Europe, North America, and China, with a male-to-female ratio of approximately 3:1, suggesting a higher susceptibility in men compared to women.^[2]^ The most commonly used medications for treating AS currently are disease modifying antirheumatic drugs (DMARDs) as well as nonsteroidal anti-inflammatory drugs (NSAIDs).^[3]^ These drugs can alleviate inflammation, ease pain in the sacroiliac joints and backbone, which are also helpful in delaying the progression of AS. However, some patients do not respond positively to these medications or develop drug tolerance, causing the efficacy of the drugs to diminish over time, potentially leading to severe infections.^[4]^ tumor necrosis factor inhibitors is one of the viable treatment options for AS, but their high rate of adverse reactions and expensive cost makes it less preferred by patients.^[5]^

AS is classified as a “Bi syndrome” in traditional Chinese medical science, which is believed to be internally caused by an innate yang deficiency in the body, as well as insufficiency of kidney and liver yin essence and a deficiency of the Du meridian, with a prevalence of cold with wind-dampness pathogen as the external cause.^[6]^ Acupuncture is a widely used and established therapeutic procedure in traditional Chinese medicine, offering benefits such as low adverse responses, significant curative effect, ease of use, and low cost. Acupuncture has been demonstrated in studies to have anti-inflammatory and analgesic properties, as well as enhanced circulation and immunological regulation; it also has a considerable therapeutic effect on arthralgia.^[7]^

Combining therapy of acupuncture and Western medicine is frequently employed in the treatment of AS, and researches have shown that acupuncture treatment can effectively improve clinical symptoms such as morning stiffness and spinal mobility, as well as improving patients’ quality of life.^[8]^ However, relevant research has not investigated whether the combination of acupuncture and Western medicine offers better clinical outcomes than Western medicine alone in treating AS. Therefore, this meta-analysis and systematic review aim to assess the clinical safety and efficacy of combined acupuncture and Western medicine therapy in treating AS, with the goal of providing a reference for clinical practice.

## 2. Methods

The Preferred Reporting Items for Systematic Reviews and Meta-Analyses (PRISMA 2020) declaration was adhered to in this systematic review.^[9]^ The protocol for this review was registered in the PROSPERO (Registration number: CRD42024509243)

### 2.1. Inclusion and exclusion criteria

#### 2.1.1. Inclusion criteria

All randomized controlled trials (RCTs) assessing the clinical effectiveness of acupuncture in conjunction with western medicine for the treatment of AS; no restriction on language or form of publication; the study population was patients with a clear diagnosis of AS, regardless of age, gender, and ethnicity; and the trial intervention consisted of acupuncture alone in conjunction with Western medical care. The control intervention was western medicine treatment alone; the outcome evaluation indexes included the overall clinical effectiveness rate (the assessment standards for AS were derived from the AS Treatment Guidelines’ analysis of clinical efficacy),^[10]^ clinical symptom measurements including thoracic mobility, the bath ankylosing spondylitis functional index (BASFI), and the bath ankylosing spondylitis disease activity index (BASDAI),^[11]^ and the visual analog scale (VAS) for pain (VAS) to quantify pain, evaluation of adverse reaction rates and values of laboratory indicators, erythrocyte sedimentation rate (ESR) and C-reactive protein (CRP).

#### 2.1.2. Exclusion criteria

No clear diagnosis; non-RCT articles; interventions in the control group were added with other treatments in addition to western medication; the experimental group was added with other treatments in addition to acupuncture in conjunction with western medication; duplication of the literature with a 0 score using the modified Jadad score; implausible outcome metrics; and studies that did not provide basic information about the subjects or interventions related to the study.

### 2.2. Search strategy

We searched China National Knowledge Infrastructure, Chinese Biomedical Literature Database (CBM), Wanfang, Chinese Science and Technology Journal Database (VIP), PubMed, Embase, and Cochrane Library for all RCTs of western medication and acupuncture combined for AS and used a combination of subject and free words to search for the following terms: “ankylosing spondylitis”, “AS”, “acupuncture”, and “acupuncture therapy.” The search period was from January 1, 2000, to July 31, 2024. We also checked the reference sheets for conference proceedings and associated papers. Table S1, Supplemental Digital Content, https://links.lww.com/MD/O991 provides the all database search approach.

### 2.3. Literature search and extraction of data

Literature was searched according to the identified search strategy, and the retrieved literature was imported into Endnote 20. Duplicates were removed, and literature was excluded by reading the title and abstract and the type of literature, e.g., studies not related to duplicates, studies not related to AS, studies on concomitant therapies, animal experiments, literature reviews, and case reports. After the initial selection, the full text of the remaining literature was retrieved. By 2 independent researchers reading the entire text in accordance with the inclusion and exclusion criteria, the literature was secondary screened. Then, 2 researchers recorded the basic data, using Excel software. Cross-checking the findings of the screening and extraction process allowed for inconsistencies to be discussed with another researcher.

### 2.4. Literature quality evaluation

Two researchers independently employed the Cochrane Collaboration Systematic Evaluator’s Manual to assess the methodological quality of the included papers, focusing on randomization procedures, randomization concealment, blinding, and bias. Disagreements among the authors of these evaluations were resolved through debate, with the assistance of another researcher (ZX), and the results were interpreted.

### 2.5. Analytical statistics

Conducted using Revman 5.4 from the Cochrane Network. Mean difference was used for measures. Count data are expressed as odds ratios or risk ratios. Both were measured with effect sizes and their 95% confidence intervals (CI). When there was heterogeneity in the meta-analysis results (defined as a test for heterogeneity *P* ≤ .1), random effects were used. The model expresses the effect, and vice versa, the fixed-effect model is used to express it. Inverted funnel analysis was utilized to test for the presence of their publication bias.

### 2.6. Evaluation of evidence quality

The grading of recommendations, assessment, development, and evaluation (GRADE) scoring technique was utilized to rate the outcome indicators’ evidence. The assessment’s material is subject to limits, inconsistencies, indirectness, imprecision, and reporting bias.

## 3. Results

### 3.1. Characteristics of included studies

**Figure 1.**
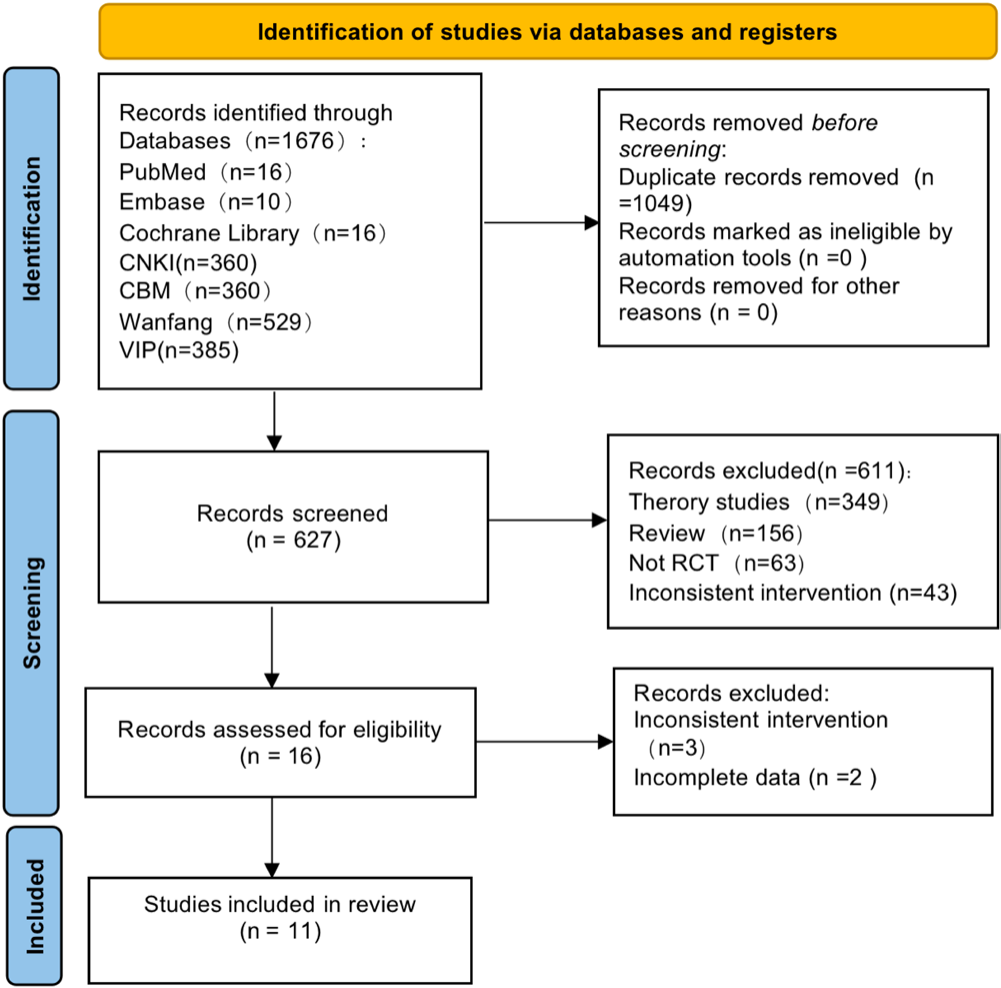
Flow chart of the process for literature retrieval.

As shown in Figure 1, 1073 citations were found using the original search strategy. On the other hand, 689 entries were deleted after the initial review, and 749 records were removed because of duplication. Out of the 16 candidate papers that were still in the running, 5 were deemed unsuitable for inclusion in the meta-analysis due to their inconsistency in interventions and lack of data.

The 11 included studies^[12–22]^ had sample sizes ranging from 40 to 116 and were all single-center investigations. With the exception of 1 case^[13]^ where the individuals’ ages were not specified, all of the subjects were adults. For AS, all research contrasted Western medication alone with acupuncture in conjunction with it. Tables 1 and 2 display the basic features and particular interventions of the included studies.

**Table 1:** Characteristics of the included studies.

Study	Sample size (treatment/control groups)	Patient age (yr)		Course of treatment (d)	Outcomes
		Treatment group	Control group		
Li 2021^[12]^	52/52	27.1 ± 2.61	26.92 ± 2.57	60	①⑥⑨
Wu 2013^[13]^	22/21	Unknown		48	④⑤⑥
Xu 2019^[14]^	30/30	40.42 ± 11.37	40.07 ± 11.56	90	①⑥⑦⑧
Liu 2019^[15]^	40/40	36.0 ± 5.4	36.5 ± 5.2	60	②④⑤⑦⑨
Meng 2022^[16]^	40/40	25.11 ± 1.25	24.50 ± 1.23	90	①②③④⑦⑧
Chen 2011^[17]^	19/21	33.68 ± 6.028	33.52 ± 5.537	90	①②⑥⑦⑧
Wu 2023^[18]^	39/39	40 ± 7	38 ± 5	21	①②③④⑤⑦⑨
Zhao 2023^[19]^	29/29	34.97 ± 11.31	33.33 ± 10.70	30	①③④⑥⑦⑧⑨
Wang 2022^[20]^	45/45	Male (57.78)/female (42.22)	Male (60.00)/female (40.00)	56	①③⑦⑧
Wang 2020^[21]^	60/56	21.19 ± 3.27	30.14 ± 5.71	21	①⑥⑨
Wen 2023^[22]^	30/30	18 to 46 (26.03 ± 8.04)	19 to 44 (25.05 ± 8.02)	84	①④⑤⑥⑦⑧⑨

① effective rate; ② thoracic mobility; ③ Schober test; ④ bath ankylosing spondylitis disease activity index; ⑤ bath ankylosing spondylitis functional index; ⑥ visual analogue scale; ⑦ C-reactive protein; ⑧ erythrocyte sedimentation rate; ⑨ adverse reactions.

**Table 2:** Characteristics of the interventions.

Study	Interventions of treatment group		Interventions of control group
	Acupuncture	Western medicine	Western medicine
Li 2021^[12]^	Acupoint selection: DU2, DU6, DU7, DU8, DU9, DU10, DU11, need to be stabbed obliquely for about 20 mm. DU3, DU4 and DU5, need to be stabbed straightly for about 15 mm. DU12, DU13 and DU14, need to be stabbed obliquely about 25 mm. DU20, flat thorn is about 20 mm. The needles were retained for 25 min, qd.	Loxoprofen sodium tablets 60 mg tid; sulfasalazine tablets 1 g bid	Loxoprofen sodium tablets 60 mg tid; sulfasalazine tablets 1 g bid
Wu 2013^[13]^	Acupoint selection:BL20, BL23, BL17, GB25, LR13, SP6, BL11, RN6, RN4, BL40, DU2. The needles were retained for 30 min and manipulated once every 15 min, qd.	Sulfasalazine tablets 0.75 g bid for minors, 1g bid for adults; Methotrexate tablets 5mg qw, 2.5 mg was added per wk to a maximum of 10 mg	Sulfasalazine tablets 0.75 g bid for minors, 1g bid for adults; Methotrexate tablets 5 mg qw, 2.5 mg was added per wk to a maximum of 10 mg
Xu 2019^[14]^	Acupoint selection:GB20,BL23,EX-B2, BL18, a-shi points. The needles were retained for 30 min and manipulated once every 10 min, qd.	Sulfasalazine tablets 1 g bid	Sulfasalazine tablets 1 g bid
Liu 2019^[15]^	Acupoint selection:Hua tuo Jia ji xue(T1-L5), BL23, BL25, DU14, BL54, GB30, a-shi points, BL40, qd.	Diclofenac sodium sustained-release tablets 75 mg qd; sulfasalazine tablets 1 g for the first wk, 2 g for the next weeks	Diclofenac sodium sustained-release tablets 75 mg qd; sulfasalazine tablets 1 g for the first wk, 2 g for the next weeks
Meng 2022^[16]^	Acupoint selection:GB20, a-shi points, EX-HN02, BL23, BL18. The needles are retained for 40 min after obtaining Qi and manipulated once a day.	Thalidomide tablets 50 mg tid	Thalidomide tablets 50 mg tid
Study	Interventions of treatment group		Interventions of control group
	Acupuncture	Western medicine	Western medicine
Chen 2011^[17]^	Acupoint selection:RN12, RN10, RN06, RN04, RN03, KI13, SP15, ST26, ST24. The needles are retained for 30 min after obtaining Qi and manipulated once every 10 min. Take 1 day off after 6 consecutive days of treatment.	Diclofenac sodium sustained-release tablets 75 mg qd; sulfasalazine tablets 0.5 g tid for the first wk, 0.75 g tid for the second wk, 1 g tid for the third and remaining weeks	Diclofenac sodium sustained-release tablets 75 mg qd; sulfasalazine tablets 0.5 g tid for the first wk, 0.75 g tid for the second wk, 1 g tid for the third and remaining weeks
Wu 2023^[18]^	Acupoint selection：KI06, BL62. After obtaining qi, the patient was instructed to perform active trunk flexion, extension, lateral bending and rotation, the movements should be slow and gentle, and the degree of activity should be as much as the patient’s pain tolerance, 15 times in each direction, during which the operator continues to manipulate needle and obtains qi. After the treatment, select BL23 and BL25, The needles were retained for 20 min, qd.	Indomethacin sustained-release capsule 75 mg qd; sulfasalazine tablets 1 g bid	Indomethacin sustained-release capsule 75 mg qd; sulfasalazine tablets 1 g bid
Zhao 2023^[19]^	Acupoint selection：TSBh1-8,9,10, (bilaterally), RFh1-6, RFh2-6, DXh2-6,8,11 (bilaterally), DWSg (bilaterally), DNsz (bilaterally) and DZBh1-5 (bilaterally). Locate and needle according to“8”circle method with the sequence as follows: TSBh1-8,9,10, (dextrally)→ RFh1-6→RFh2-6→DXh2-6,8,11, (sinistrally)→DNsz (sinistrally)→DZBh1-5 (dextrally) →DZBh1-5 (sinistrally)→DNsz (dextrally)→DXh2-6,8,11 (dextrally)→DWSg (sinistrally)→TSBh1-8,9,10 (sinistrally). The needles are retained for 30 min after obtaining Qi and manipulated every other day(qod)	Celecoxib 0.2 g bid	Celecoxib 0.2 g bid
Study	Interventions of treatment group		Interventions of control group
	Acupuncture	Western medicine	Western medicine
Wang 2022^[20]^	Acupoint selection: Referring to Xue Ligong “Chinese Meridians and Tendons,” select 8 to 10 knotty tendon focal point of solar plexus tendon for treatment. The needles were retained for 30 min, qd, 5 consecutive days of treatment and 2 d of rest.	Sulfasalazine tablets 1 g qd for the first week, 1g bid for the second week, 1.5 g bid for the third week	Sulfasalazine tablets 1 g qd for the first week, 1 g bid for the second week, 1.5 g bid for the third week
Wang 2020^[21]^	Acupoint selection: 6 to 8 acupoints downward the 7th cervical spinous process of the thoracolumbar part of the Du Meridian with an interval of about 4 to 5 cm. Needling: oblique Insertion 2 to 2.5 cm at an angle of 45°, accompanying with a-shi points. The needles are retained for 30 min after obtaining Qi and manipulated once a d, 6 times a wk.	Diclofenac sodium sustained-release tablets 75 mg qd; sulfasalazine tablets 1 g tid	Diclofenac sodium sustained-release tablets 75 mg qd; sulfasalazine tablets 1 g tid
Wen 2023^[22]^	Acupoint selection：DU03, DU04, DU14, BL18, BL23, EX-B02. Categorizing acupuncture points into 3 divisions: celestial, human, and earth (shallow, medium, and deep)，operate according to the “one back, 3 forward, drill and pick 4 sides” technique. Like a turtle in the earth, it probes again and again, deeper and deeper, drilling and picking in all directions. The needles were retained for 30 min, qd, 5 consecutive days of treatment and 2 d of rest.	Thalidomide tablets 75 mg qd; diclofenac sodium double-release enteric capsule 75 mg bid	Thalidomide tablets 75 mg qd; diclofenac sodium double-release enteric capsule 75 mg bid

bid = twice a day, BL = bladder meridian, DU = du meridian, EX = extraordinary, GB = gallbladder meridian, KI = kidney meridian, LR = liver meridian, qd = once a day, qod = every other day, qw = once a week, RN = ren meridian, SP = spleen meridian, ST = stomach meridian, tid = 3 times a day.

### 3.2. Risk of bias assessment

**Figure 2.**
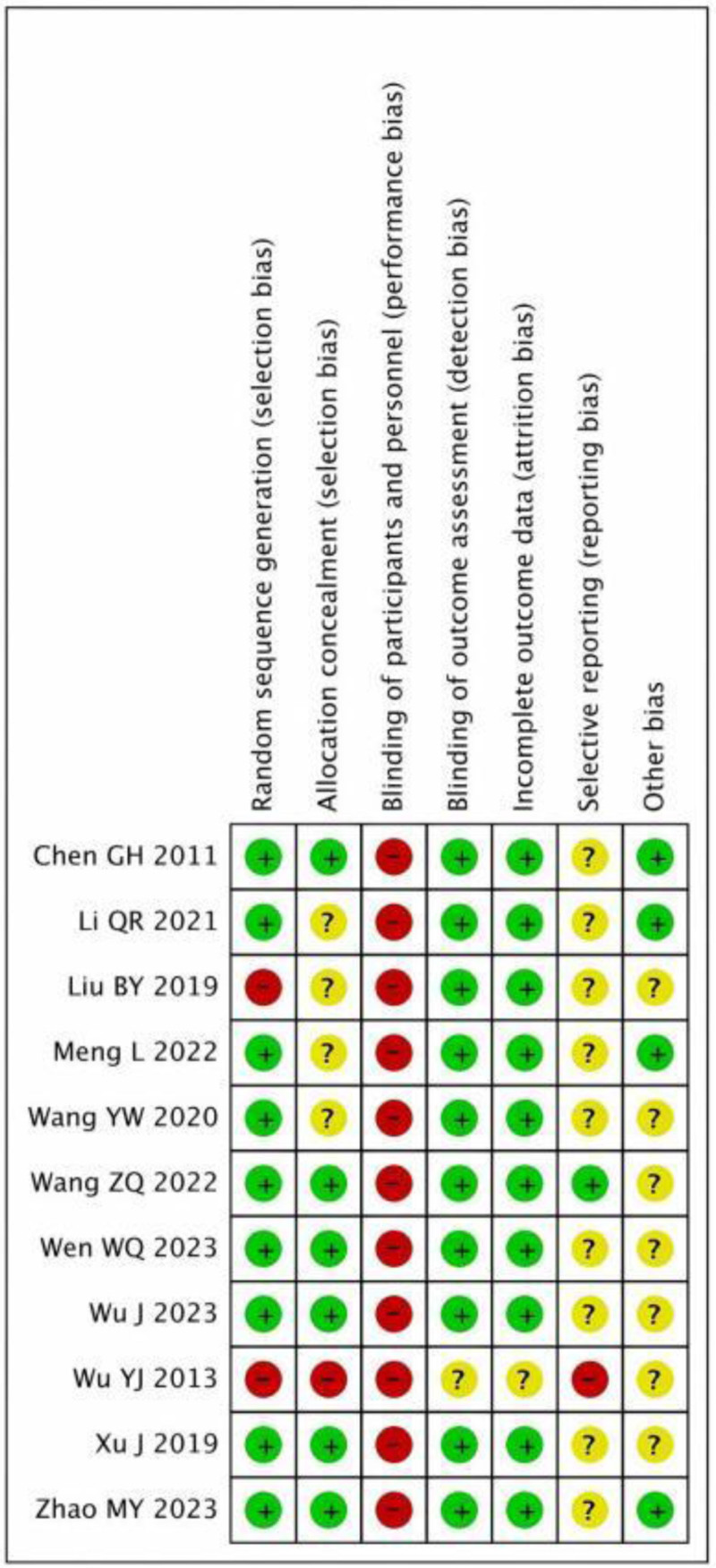
Risk assessment chart of bias in included studies.

There was no detailed description of the allocation concealment strategies in any of the investigations.^[12–22]^ A study^[13]^ was found to have a high potential for bias due to the absence of comprehensive explanations of the statistical procedures. Figure 2 displays the results of the risk of bias evaluation.

### 3.3. Outcome measurements

#### 3.3.1. Effective rate

**Figure 3.**
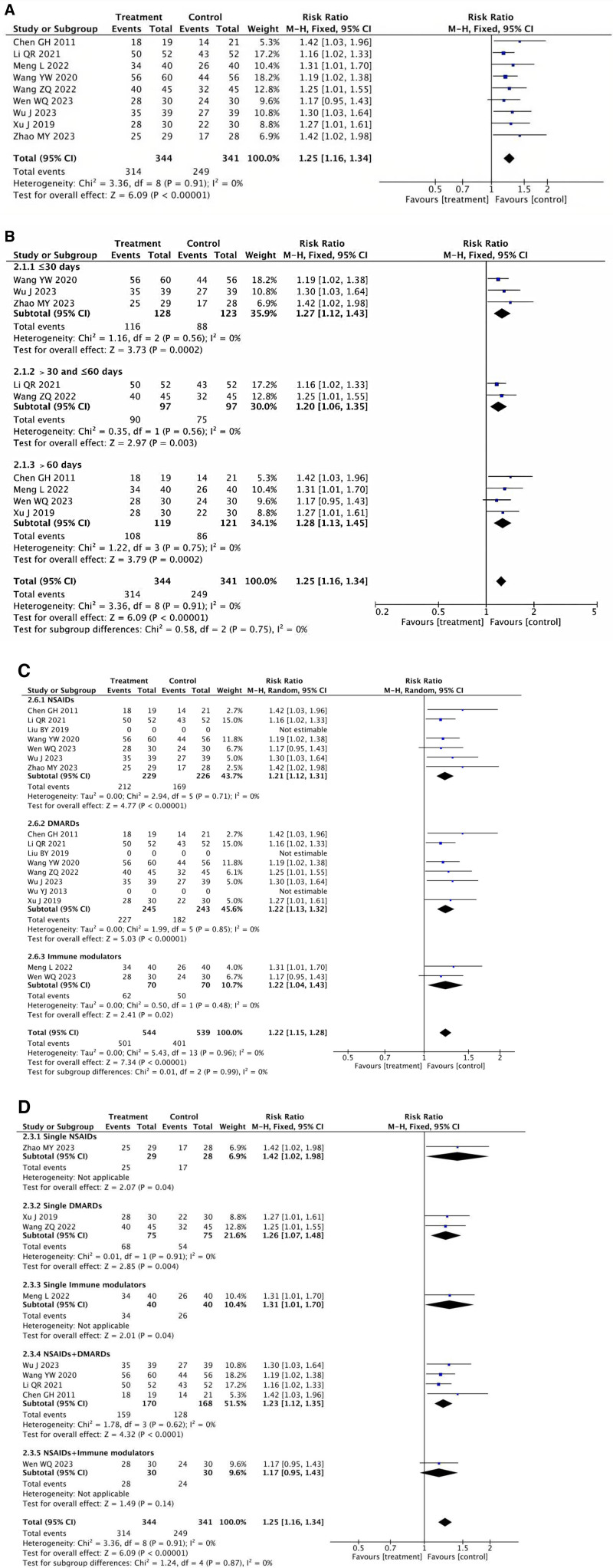
(A) Meta-analysis of effective rate between the treatment group and the control group. (B) Meta-analysis of effective rate in subgroups between the treatment group and the control group. (C) Meta-analysis of effective rate in subgroups between the treatment group and the control group. (D) Meta-analysis of effective rate in subgroups between the treatment group and the control group.

Nine RCTs^[12,14,16–22]^ involving 685 patients reported the effective rate of acupuncture treatment for AS, with results of *I*^2^ = 0% and *P* < .00001, indicating no heterogeneity. As shown in Figure 3A, the fixed-effect model meta-analysis revealed a significantly higher effective rate for the treatment group [RR = 1.25, 95% CI: 1.16, 1.34, *P<*0.00001]. In total 3 categories were created for the participants according to the length of treatment (≤30 days, >30 days, and < 60 days, >60 days). Despite the wide variations in treatment courses between the 3 studies, there was no discernible difference between the subgroups (≤30 days: *P* = .56, *I*^2^ = 0%; >30 days and ≤ 60 days: *P* = .56, *I*^2^ = 0%; >60 days: *P* = .75, *I*^2^ = 0%). As demonstrated in Figure 3B, the analysis’s conclusions revealed that each grouping performed better than the control (≤30 days: [RR = 1.27，95% CI: 1.12–1.43, *P* = .0002]: > 30 and ≤ 60 days: [RR = 1.20, 95% CI: 1.06–1.35, *P* = .003]: >60 days: [RR = 1.28, 95% CI: 1.13–1.45, *P* = .0002]).

In addition, we also categorized them into 3 classes based on the sorts of Western medications: Immune modulators, DMARDs, and NSAIDs. The results showed that each subgroup was more effective than the control group (NSAIDs: [RR = 1.24, 95% CI: 1.14–1.35, *P* < .00001]; DMARDs: [RR = 1.24, 95% CI: 1.14–1.34, *P* < .00001]; Immune modulators: [RR = 1.24, 95% CI: 1.05–1.47, *P* = .01]). This was because there was no significant heterogeneity among the 3 subgroups (*P* < .00001, *I*^2^ = 0%) (Fig. 3C).

Based on various Western medication combinations, we separated the control group into 5 subgroups: Single NSAIDs, Single DMARDs, Single Immune modulators, NSAIDs + DMARDs, NSAIDs + DMARDs, and NSAIDs + Immune modulators. Because there were no significant differences among the 5 subgroups(*P* < .00001, *I*^2^ = 0%), we used a fixed-effect model, and the results indicated each subgroup showed greater efficacy than the control group. (Single NSAIDs: [RR = 1.42,95% CI = 1.02–1.98, *P* = .04]; single DMARDs: [RR = 1.26, 95% CI = 1.07–1.48,*P* = .004]; single immune modulators: [RR = 1.31, 95% CI = 1.01–1.70, *P* = .04]; NSAIDs + DMARDs: [RR = 1.23, 95% CI = 1.12–1.35,*P* < .0001]; NSAIDs + immune modulators: [RR = 1.17, 95% CI = 0.95–1.43, *P* = .14] (Fig. 3D).

#### 3.3.2. Thoracic mobility

**Figure 4.**
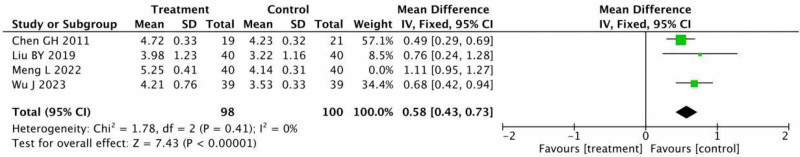
Meta-analysis of thoracic mobility between the treatment group and the control group.

Four RCTs^[15–18]^ evaluated thoracic mobility in 278 patients. The initial analysis indicated significant heterogeneity (*P* < .0001, *I*^2^ = 88%). Upon applying a 1-by-1 elimination strategy to identify the source of heterogeneity, it was found that excluding the study by Meng L (2022) resulted in no heterogeneity (*P* = .41, *I*^2^ = 0%), as depicted in Figure 4. A subsequent fixed-effect model meta-analysis revealed enhanced mobility in the treatment group [MD = 0.58, 95% CI = 0.43–0.73, *P*<0.00001].

#### 3.3.3. VAS for pain

**Figure 5.**
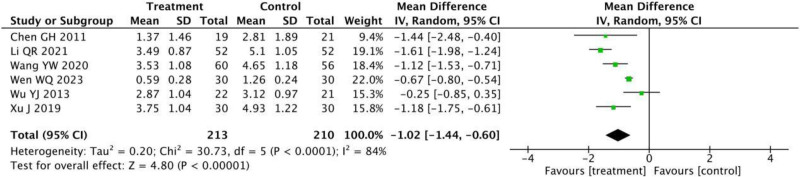
Meta-analysis of visual analogue scale for pain between the treatment group and the control group.

VAS for pain was recorded by 423 patients in 6 RCTs.^[12–14,17,21,22]^ Significant heterogeneity was shown by *P* < .0001 and *I*^2^ = 84% of the data (Fig. 5). Given that the heterogeneity persisted regardless of the exclusion of any single study, a random-effects model meta-analysis was conducted, demonstrating a significant reduction in VAS scores in the treatment group compared to the control group (MD = –1.02, 95% CI = −1.44 to −0.60, *P* < .00001).

#### 3.3.4. Bath ankylosing spondylitis functional index

**Figure 6.**
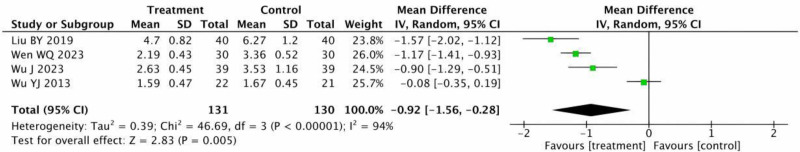
Meta-analysis of BASFI between the treatment group and the control group. BASFI = bath ankylosing spondylitis functional index.

Four RCTs^[12,13,18,22]^ reporting BASFI involved a total of 261 patients. As shown in Figure 6, the heterogeneity was substantial (*P* < .00001, *I*^2^ = 94%). The heterogeneity remained unaffected by the removal of any study, suggesting a different factor unrelated to the outcomes was responsible for the difference. A random-effects model meta-analysis indicated that the treatment group had significantly lower BASFI scores than the comparison group (MD = –0.92, 95% CI = −1.56 to −0.28, *P* = .005).

#### 3.3.5. Bath ankylosing spondylitis disease activity index

**Figure 7.**
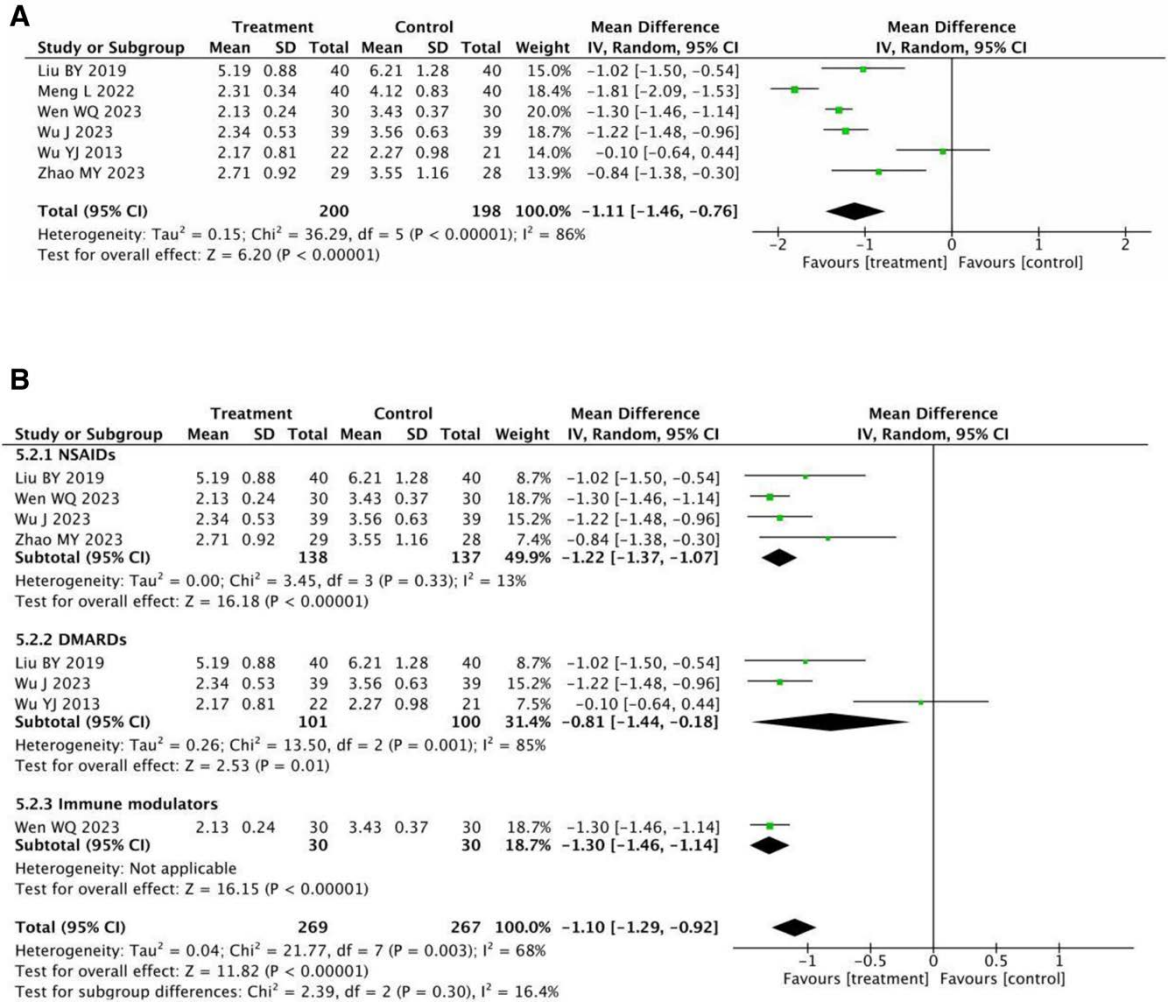
(A) Meta-analysis of BASDAI between the treatment group and the control group. (B) Meta-analysis of BASDAI in subgroups between the treatment group and the control group. BASDAI = bath ankylosing spondylitis disease activity index.

Based on 6 RCTs^[13,15,16,18,19,22]^ with 398 patients who reported BASDAI, a considerable variation between studies was observed in the heterogeneity test results (*P* < .00001, *I*^2^ = 86%), as seen in Figure 7A. The examination of heterogeneity’s findings were unaffected by the removal of any research, suggesting that the heterogeneity was an independent component. Consequently, the meta-analysis employed a random-effect model. The figure can be observed a statistically significant difference in the BASDAI scores between the 1 in the therapy group and the 1 used as a control (MD = –1.11, 95% CI: −1.46 to −0.76, *P* < .00001).

We classified patients into 3 subgroups according on their choice of western medication type: NSAIDs, DMARDs, and Immune modulators (see Fig. 7B). The studies were highly heterogeneous (*P* < .00001, *I*^2^ = 68%), and we used a random-effects model to show that each subgroup was more effective than the control group (NSAIDs: [MD = –1.22, 95% CI = −1.37 to –1.07, *P* < .00001]; DMARDs: [MD = –0.81, 95% CI = −1.44 to −0.18, *P* = .01]; immune modulators: [MD = –1.30, 95% CI = −1.46 to –1.14, *P* < .00001]).

#### 3.3.6. Schober test

**Figure 8.**
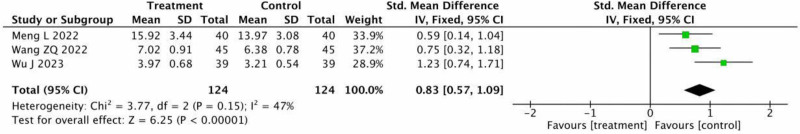
Meta-analysis of Schober test between the treatment group and the control group.

The Schober test was recorded by 248 patients in 3 RCTs.^[15,18,20]^ The findings showed no significant heterogeneity, with *P* = .15 and *I*^2^ = 47%. The meta-analysis results indicated that the treatment group’s Schober test scores improved more than the control group’s (SMD = 0.83, 95% CI: 0.57–1.09, *P* < .00001), which is consistent with our fixed-effect hypothesis (Fig. 8).

#### 3.3.7. Erythrocyte sedimentation rate

**Figure 9.**
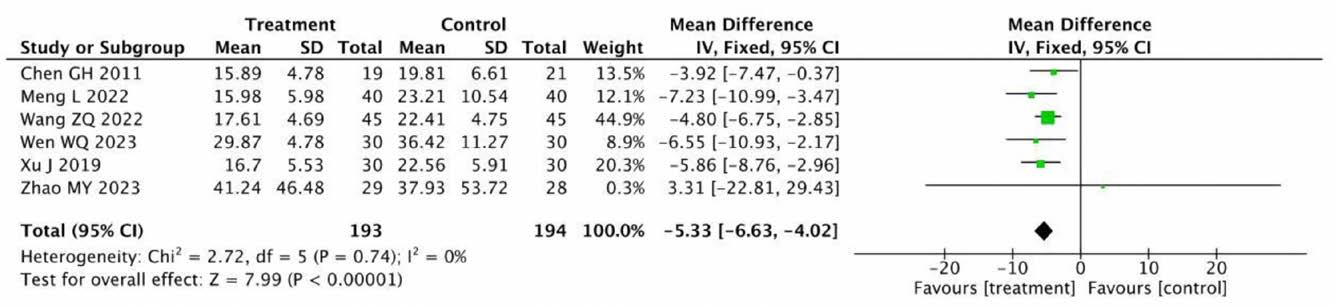
Meta-analysis of ESR between the treatment group and the control group. ESR = erythrocyte sedimentation rate

Six RCTs^[14,16,17,19,20,22]^ with 387 participants recorded ESR, which showed no study heterogeneity (*P* = .74, *I*^2^ = 0%). The treatment group’s ESR levels were statistically significantly reduced than those of the control group, according to the results of a fixed-effect model meta-analysis. (MD = –5.33, 95% CI = −6.63 to −4.02, *P* < .00001) (Fig. 9).

#### 3.3.8. C-reactive protein

**Figure 10.**
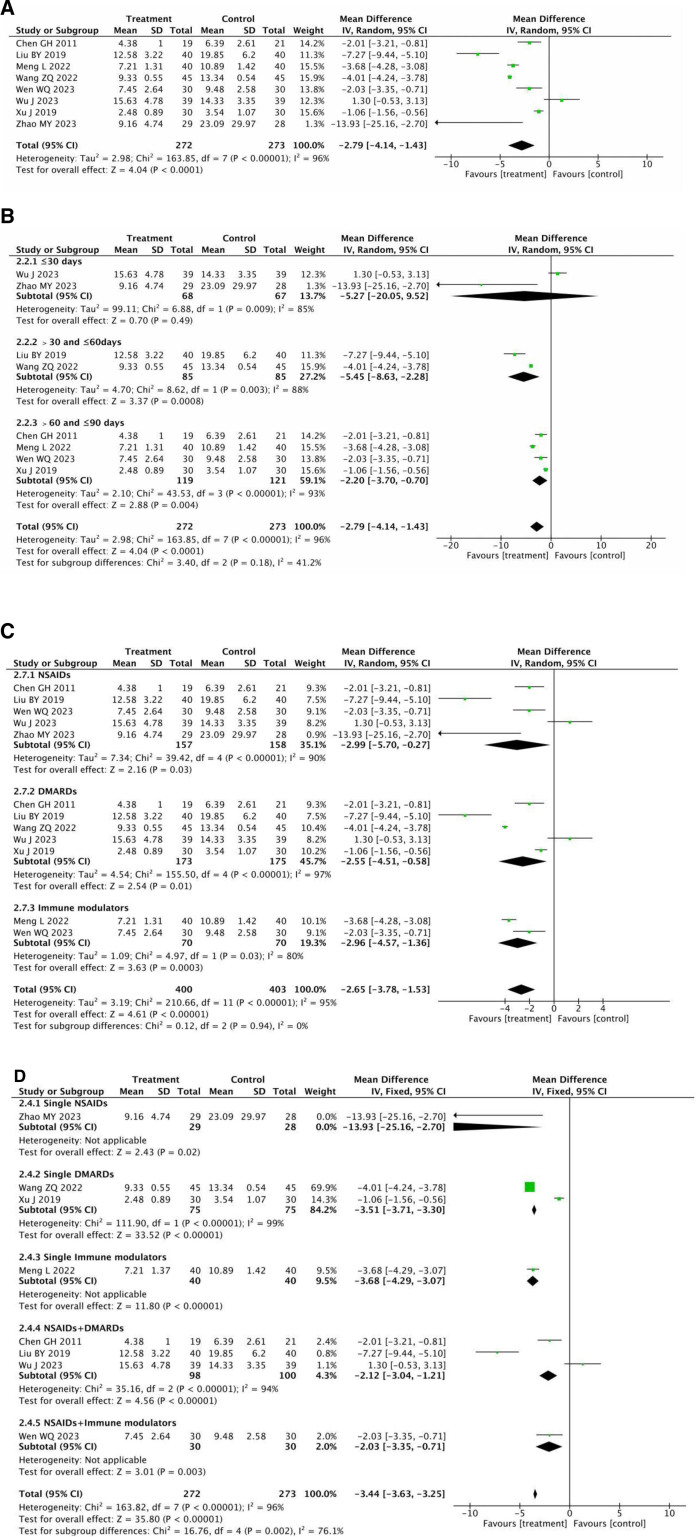
(A) Meta-analysis of CRP between the treatment group and the control group. (B) Meta-analysis of CRP of subgroups between the treatment group and the control group. (C) Meta-analysis of CRP of subgroups between the treatment group and the control group. (D) Meta-analysis of CRP of subgroups between the treatment group and the control group. CRP = C-reactive protein.

CRP was reported in 8 RCTs^[14–20,22]^ involving 545 individuals. As seen in Figure 10A, the heterogeneity was significance (*P* < .00001, *I*^2^ = 96%), and it was not completely removed even though we performed a subgroup analysis according to therapy duration. After analyzing these 8 texts, It was found that the various Western medications, dosages, acupuncture points, and numbers could be related to the cause of heterogeneity. The results of a meta-analysis using a random-effect model indicated that the CRP levels in the treatment group were lower than those in the matched control group (MD = –2.79, 95% CI = −4.14 to −1.43, *P* < .0001).

Due to how long the treatment lasted, it was split into 3 subgroups: <30 days, >30 and ≤ 60 days, and > 60 days. According to the random effects analysis, the 3 treatment subgroups that remained had lower CRP levels than the control subgroups, with the exception of the < 30 days grouping, which did not show any statistical significance (>30 and ≤ 60 days: [MD = –5.45, 95% CI = −8.63 to −2.28, *P* = .0008]; >60 days: [MD = –2.20, 95% CI = −3.70 to −0.70, *P* = .004]) (Fig. 10B).

Because the choice of western drugs may influence clinical efficacy, and CRP is an important evaluation indicator of clinical effectiveness, we divided them into 5 subgroups based on the various choices of western drugs in the literature: single NSAIDs, single DMARDs, single Immune modulators, NSAIDs + DMARDs, NSAIDs + DMARDs, and NSAIDs + Immune modulators. We employed a random-effects model to account for considerable heterogeneity among the 5 subgroups (*P* < .00001, *I*^2^ = 96%), and found that each subgroup outperformed the control group(Single NSAIDs: [MD = –13.93, 95% CI = −25.16 to –2.70, *P* = .02]; Single DMARDs: [MD = –2.54, 95% CI = −5.43 to −3.08, *P* = .08]; single immune modulators: [MD = –3.68, 95% CI = −4.28 to −3.08, *P* < .00001]; NSAIDs + DMARDs: [MD = –2.61, 95% CI = −6.73 to 1.51,*P* < .00001]; NSAIDs + immune modulators: [MD = –2.03, 95% CI = −3.35 to −0.71,*P* = .003] (see Fig. 10C).

We further separated them into 3 categories based on the kind of Western medications (NSAIDs, DMARDs, and immune modulators) in Figure 10D. We employed a random-effects model due to the considerable heterogeneity among the 3 subgroups (*P* < .00001, *I*^2^ = 95%). NSAIDs: [MD = –2.99, 95% CI = −5.70 to –0.27, *P* = .03]; DMARDs: [MD = –2.55, 95% CI = −4.51 to −0.58, *P* = .01]; immune modulators: [MD = –2.96, and 95% CI = −4.57 to –1.36, *P* = .0003]; all subgroups were shown to be more effective than the control group.

#### 3.3.9. Adverse reactions

**Figure 11.**
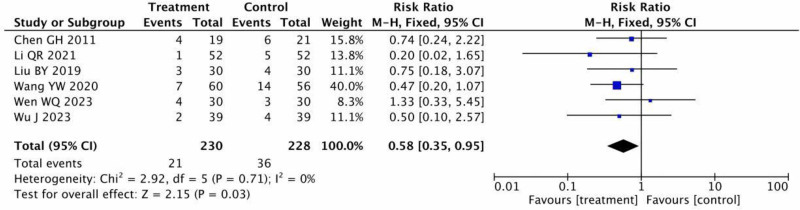
Meta-analysis of adverse reactions between the treatment group and the control group.

The rate of adverse responses was reported from 6 RCTs^[12,15,17,18,21,22]^ involving 458 participants. The results of *P* = .71 and *I*^2^ = 0% indicated no significant heterogeneity, as shown in Figure 11. A meta-analysis utilizing a fixed-effect model showed that the therapy group experienced fewer adverse effects [RR = 0.58, 95% CI = 0.35–0.95, *P* = .03].

### 3.4. Publication bias

**Figure 12.**
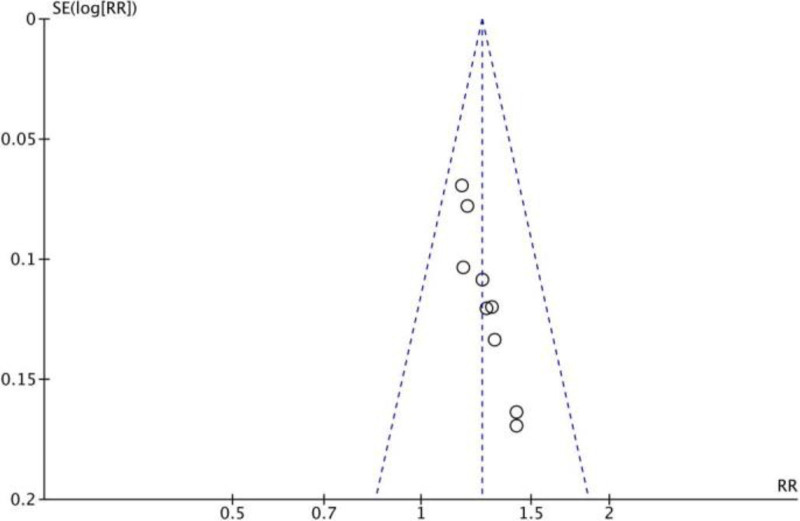
Funnel plot of publication bias.

An asymmetric funnel plot was found when it was utilized to search for publication bias in outcome indicators, which could indicate the presence of publication bias (Fig. 12). Given that all included studies were small-sample Chinese controlled trials with predominantly positive results in the literature, the absence of negative results may indicate publication bias.

### 3.5. Sensitivity analysis

**Figure 13.**
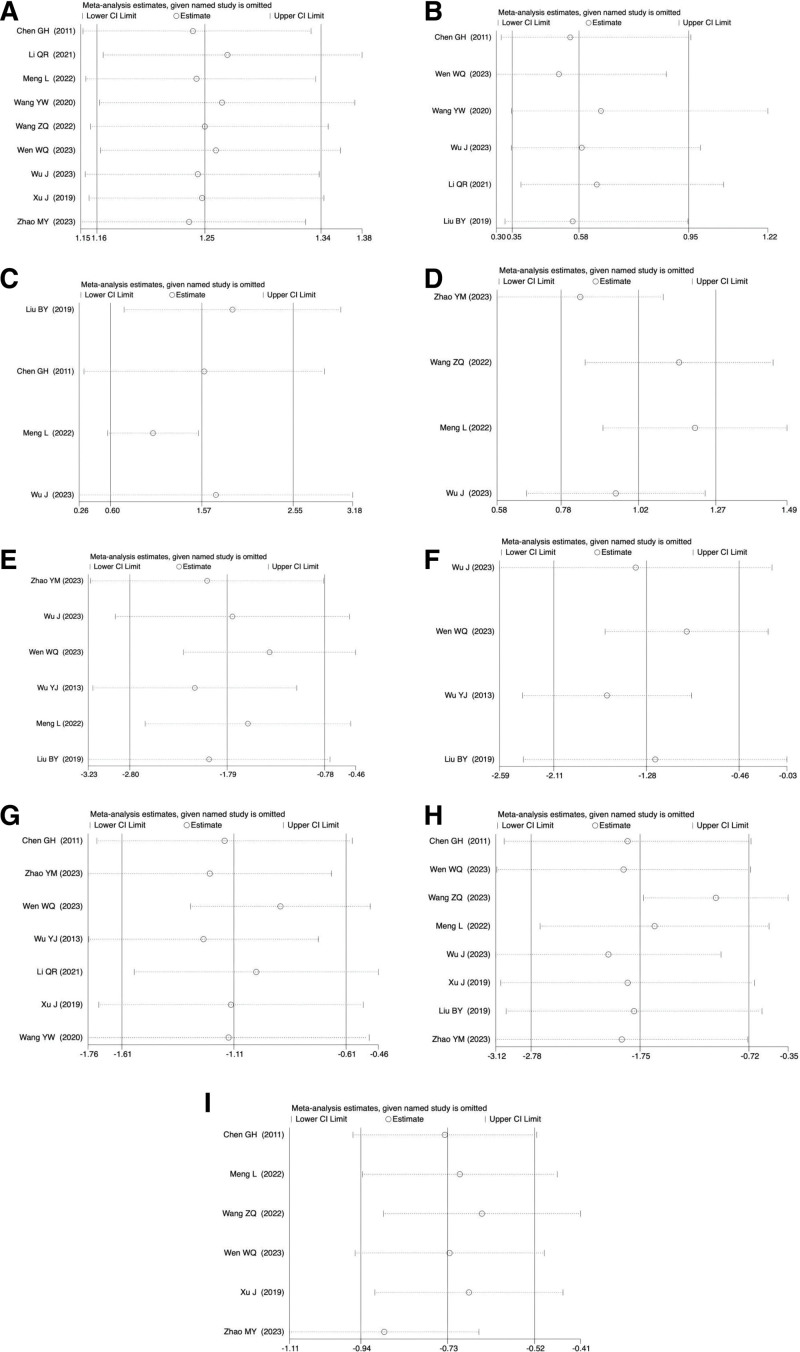
(A) Sensitivity analysis of effective rate. (B) Sensitivity analysis of adverse reactions. (C) Sensitivity analysis of thoracic mobility. (D) Sensitivity analysis of Schober test. (E) Sensitivity analysis of BASDAI. (F) Sensitivity analysis of BASFI. (G) Sensitivity analysis of visual analogue scale for pain. (H) Sensitivity analysis of C-reactive protein. (I) Sensitivity analysis of erythrocyte sedimentation rate. BASDAI = bath ankylosing spondylitis disease activity index, BASFI = bath ankylosing spondylitis functional index.

Using the process of 1-by-1 elimination, a sensitivity analysis of the aforementioned indicators was conducted. Even after excluding any study, the combined findings of the remaining trials were statistically significant, as shown in Figure 13, suggesting that the meta-analysis’s conclusions were solid.

### 3.6. Overall quality of evidence by GRADE

**Figure 14.**
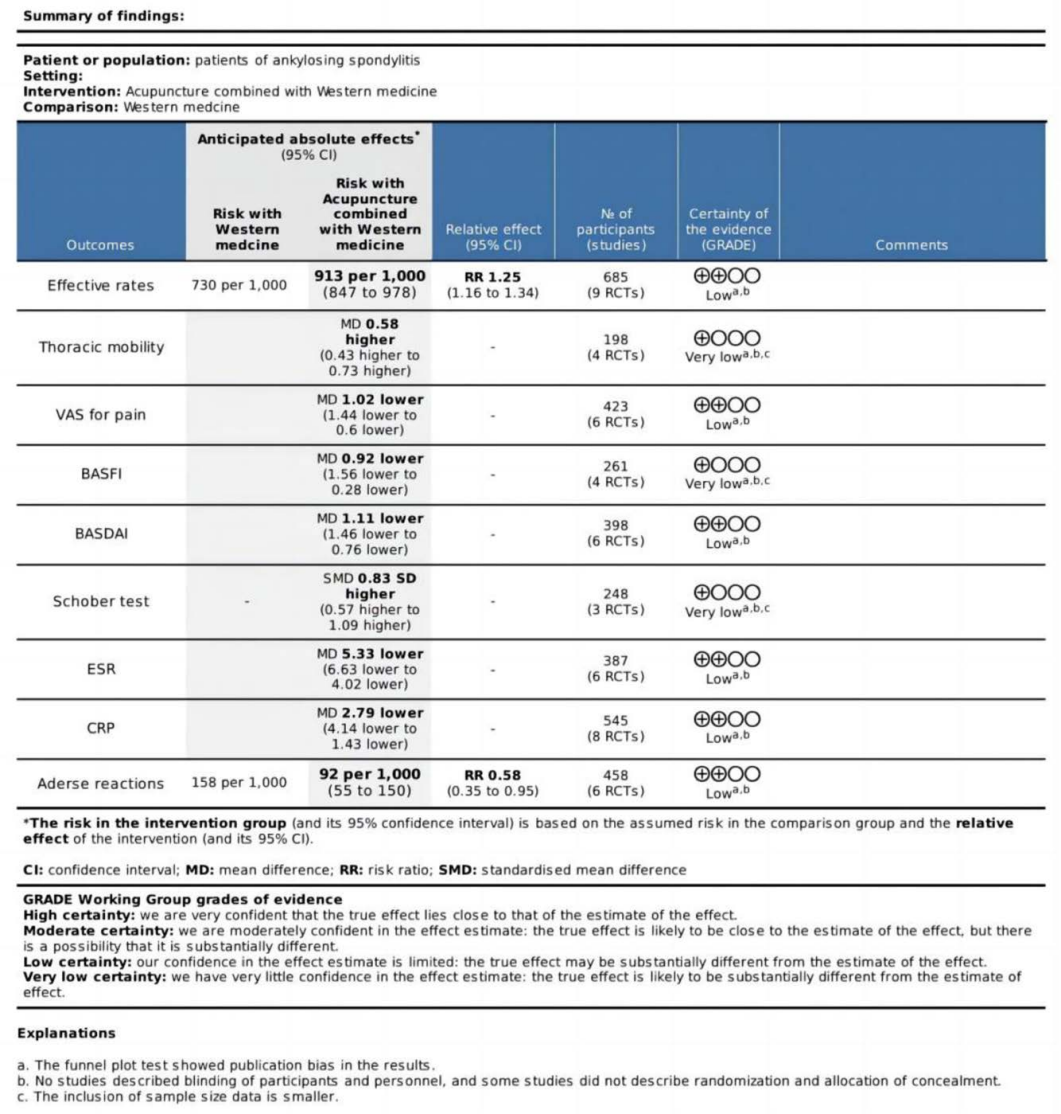
Evidence quality evaluation results.

The GRADE approach was used to assess each outcome indicator’s quality (Fig. 14). There was “low” quality evidence for the effective rate, ESR, CRP, VAS, BASDAI, and adverse reactions. The degrading factors included: a. The funnel plot test showed publication bias in the results. b. No studies described blinding of participants and personnel, and some studies did not describe randomization and allocation of concealment. The BASFI, thoracic mobility, and Schober test evidence quality was rated as “very low.” Degrading factors included: a. The funnel plot test showed publication bias in the results. b. No studies described blinding of participants and personnel, and some studies did not describe randomization and allocation of concealment. c. The inclusion of sample size data is smaller.

## 4. Discussion

In this study, a total of 809 individuals from 11 RCTs combining Western medicine and acupuncture were examined. The findings demonstrated that the combination of acupuncture and Western medicine in the diagnostic therapy of AS showed greater benefits and therapeutic effects compared to Western medicine alone. Eight clinical diagnostic indicators of AS were analyzed in this study, including total effective rate, CRP, ESR, BASDAI score, BASFI score, Schober test, thoracic motion, and VAS score.

Inflammation is the core pathological change in the course of AS disease. CRP and ESR play crucial roles in diagnosing and evaluating the activity of AS disease.^[23]^ This study included 8 RCT trials evaluating changes in CRP and 6 RCT trials evaluating changes in ESR, indicating that the combination of acupuncture and Western medicine therapy is more effective in anti-inflammatory treatment. Acupuncture exerts its anti-inflammatory effects through various mechanisms, including activation of TLR/MyD88, NOD, IκBα/NF-κB, and P38 MAPK pathways, which prevent the generation of pro-inflammatory mediators and inflammasomes. Acupuncture also regulates the equilibrium of helper T cell subsets and enhances SOD activity, inhibits oxidative stress, and eliminates reactive oxygen species production.^[24]^

Pain, stiffness, and fatigue are common symptoms throughout AS, and the changes in symptoms are significantly correlated with disease activity. The VAS is a quick and sensitive tool for assessing AS, as it can reflect the pathological changes throughout the disease course.^[25]^ This study indicates that the intricate network of nerves, microvessels, and collagen fibers in acupuncture sites, as well as the enrichment of mast cells in these areas, may contribute to the efficacious treatment of AS symptoms with acupuncture. Acupuncture has the ability to distort collagen fibers, activate the TRPV channels on mast cell membranes, and stimulate the release of biologically active substances, thereby activating pain-relieving effects through the activation of neural receptors.^[26]^

In addition, the joint mobility and objective measurement indicators of patients can also reflect the disease activity from various dimensions.^[27]^ BASDAI, BASFI, Schober test, and chest expansion are all indicators of axial function evaluation. BASDAI and BASFI are scored based on patients’ subjective feelings, reflecting disease activity and physical function in AS. Results from this research suggest that using acupuncture in conjunction with Western medicine can effectively reduce BASDAI and BASFI scores, indicating that acupuncture can improve patients’ physical activity and quality of life. Schober test is used to measure lumbar spine mobility in an upright position. AS patients commonly experience limited flexion, extension, and rotation of the spine. The numerical changes in 4 RCTs evaluating Schober test suggest that acupuncture has a good improvement effect on lumbar spine mobility and normal physiological activities of the body. Due to spinal stiffness and inflammation, the chest circumference changes when patients perform deep exhalation and inhalation, indicating changes in chest expansion. Four RCTs evaluated changes in chest expansion and showed that acupuncture can effectively improve this symptom. Additionally, the incidence of adverse reactions was noted in over half of the trials, and the findings show that acupuncture in conjunction with Western treatment can successfully lower the incidence of unpleasant reactions in patients with AS.

**Figure 15.**
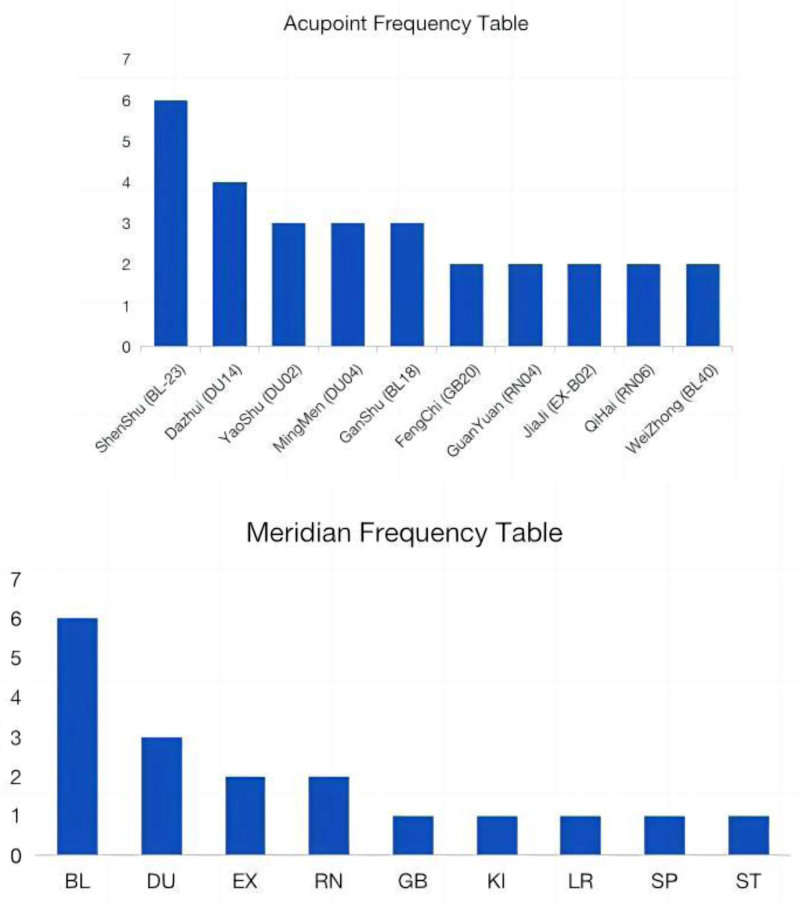
Acupoint and meridian frequency graphs.

We tallied the acupoint and meridian frequency graphs (Fig. 15) to examine the great heterogeneity they bring, since the literature in the article chose various acupoints, respectively. The frequency of using various acupoints and meridians in the treatment of AS varies significantly, as shown by the 2 graphs. Acupoints like Shenshu, Dazhui, and Mingmen are used more frequently than meridians like Bladder Meridian (BL), Du Meridian (DU), and Ren Meridian (RN), particularly acupoints of the Bladder Meridian. The discrepancies in the selection of acupuncture points may be attributed to several factors. Primarily, from Traditional Chinese Medicine perspective, these points and meridians are deemed more pertinent to the etiology of AS. This is based on the principle that “wherever a meridian passes through, it is the area that can be treated by that meridian.” Additionally, the pathogenesis of AS predominantly affects the spine and back, which is associated with the bladder meridian and the directing meridian. The onset of AS is primarily associated with the spine and back, exhibiting a close correlation with the Bladder Meridian and the Du Meridian. Consequently, the points situated on these 2 meridians are frequently selected for therapeutic intervention. In addition, the tenets of Chinese medicine posit that “noncommunication causes pain and non-glorification causes pain,” and that the obstruction of qi and blood flow can result in pain, as can insufficient qi and blood flow. The Ren and Stomach meridians are associated with the qi and blood flow of the entire body. Consequently, acupuncture on these 2 meridians is often employed to alleviate pain by regulating the body’s qi and blood flow. Furthermore, the design and sample size of different studies may influence the selection of acupuncture points and meridians, resulting in variations in their frequency of use.

In light of the aforementioned evidence, it is evident that the meridian theory of Chinese medicine and the high-frequency acupoints identified in the study, including Shenshu, Dazhui, and Mingmen, necessitate the adoption of distinct principles for acupoint selection and matching, tailored to the specific symptoms or stages of the disease. In the acute stage, the objective is to provide analgesia and reduce inflammation. Acupoints such as Dazhui and Hegu can be employed in conjunction with acupuncture and moxibustion. In the chronic stage, the objective is to bolster the body’s resilience and enhance joint mobility. Acupoints such as Shenshu and Zusanli are frequently utilized, with mild needling and moxibustion. In the treatment of back stiffness and pain, points on the bladder meridian, such as Shenshu, Dachangshu, Dazhui of the Du Meridian, may be selected as appropriate. The efficacy of the treatment can be augmented through the use of electroacupuncture. In cases of fatigue and weakness affecting the entire body, the acupoints Zusanli and Guanyuan can be employed to replenish vital energy. In instances of depression and anxiety, acupuncture points such as Neiguan and Shenmen may be employed for the purpose of regulating and adjusting the relevant physiological processes. The treatment should be evidence-based, comprehensive, and long-term to achieve optimal results.

Otherwise, our study revealed significant heterogeneity in certain outcome metrics, a finding that aligns with the results of the meta-analysis conducted by Wenjia^[28]^ and the Bayesian meta-analysis by Yi.^[29]^ Zhao study demonstrated significant heterogeneity in certain outcome metrics, including the degree of thoracic extension, finger-ground distance, occipital-wall distance, Schober test, ESR, and time to morning stiffness. In contrast, Yuan study demonstrated significant heterogeneity in certain outcome metrics, including the degree of thoracic extension, finger-ground distance, occipital-wall distance, Schober test, ESR, and time to morning stiffness. However, the specific heterogeneity values were not explicitly reported. Additionally, the evidence was evaluated as low or very low in exactness, and the comparisons, with the exception of the comparison of endothermic acupuncture versus conventional acupuncture, were downgraded due to a serious risk of bias. Moreover, some of the outcome indicators, including CRP, BASDAI, and VAS, demonstrated significant heterogeneity, which persisted even after subgroup analysis. These issues of high heterogeneity may impact the stability and interpretability of the results, thereby affecting the reliability of the conclusions. Consequently, we employed multiple subgroup analyses, sensitivity analyses, and random-effects models to address these issues, thereby enhancing the reliability and scientific validity of the findings.

### 4.1. Limitations

Our study has some limitations. Firstly, the sample size was somewhat limited, and all studies were carried out in Asian locations, which might have regional restrictions. To further generalize these findings, larger-scale RCT research with several centers will be required in the future. Secondly, blinding was not feasible in acupuncture treatment as the needles are directly inserted into skin and acupoints of the patient. This may bring about a lower authority of the study due to the inability to use blinding to prevent placebo effects or observer effects.^[30]^ Thirdly, due to the fact that AS is a chronic illness, proper care and monitoring are necessary. The long-term effectiveness of acupuncture in conjunction with Western therapy for treating AS, however, cannot be assessed because no trial included follow-up data. Lastly, the meta-analysis is heterogeneous due to the variability of the treatment procedures, inclusion and exclusion criteria, and diagnostic criteria. Although subgroup analysis and sensitivity analysis were performed, it was still not possible to avoid the confusion of statistical results caused by heterogeneity.

## 5. Conclusion

In conclusion, acupuncture combined with Western medicine appears to be a safe and effective treatment option for AS, especially in reducing clinical symptoms. Further large-sample, high-quality, continuous monitoring RCTs are needed to validate the findings of this analysis due to the low level of evidence quality in the reported trials.

## Author contributions

**Conceptualization:** Xindan Cao, Yadan Zhang, Zhihui Xiao, Jianhong Peng.

**Data curation:** Xindan Cao, Yadan Zhang, Zhihui Xiao, Jianhong Peng.

**Formal analysis:** Xindan Cao, Yadan Zhang, Zhihui Xiao, Jianhong Peng.

**Investigation:** Xindan Cao, Yadan Zhang, Jianhong Peng.

**Methodology:** Xindan Cao, Yadan Zhang, Zhihui Xiao, Jianhong Peng.

**Project administration:** Xindan Cao.

**Resources:** Xindan Cao.

**Software:** Xindan Cao, Jianhong Peng.

**Supervision:** Xindan Cao.

**Validation:** Xindan Cao.

**Visualization:** Xindan Cao.

**Writing – original draft:** Xindan Cao, Yadan Zhang, Zhihui Xiao, Jianhong Peng.

**Writing – review & editing:** Xindan Cao, Yadan Zhang, Zhihui Xiao, Jianhong Peng.

## Supplementary Material


