# Supplementary material for: Efficacy and safety of acupuncture combined with Western medicine in the treatment of ankylosing spondylitis: A systematic review and meta-analysis
Source: Medicine (Baltimore). 2025 May 23;104(21):e42468. doi: 10.1097/MD.0000000000042468 (PMC12113976; doi:10.1097/MD.0000000000042468)
Supplement: Supplementary file 1 [file medi-104-e42468-s001.docx]

Table S1

| The search strategy for PubMed. | | |  |  |  |
| --- | --- | --- | --- | --- | --- |
| number |  | Search terms |  |  |  |
| 1# | ("Acupuncture"[MeSH Terms] OR "acupuncture therapy"[MeSH Terms] | | | |  |
| 2# | "acupuncture treatment*"[Title/Abstract] OR "treatment acupuncture"[Title/Abstract] OR "therapy acupuncture"[Title/Abstract] OR "acupotom*"[Title/Abstract]) | | | | |
| 3# | 1# OR 2# | | | |  |
| 4# | ("spondylitis, ankylosing"[MeSH Terms]) | | | |  |
| 5# | "bechterews disease"[Title/Abstract] OR "marie struempell disease"[Title/Abstract] OR "spondylarthritis ankylopoietica"[Title/Abstract] OR "spondyloarthritis ankylopoietica"[Title/Abstract] OR "ankylosing spondylitis"[Title/Abstract] OR "ankylosing spondylarthritis"[Title/Abstract] OR "ankylosing spondylarthritides"[Title/Abstract] | | | | |
| 6# | 4# OR 5# | | | | |
| 7# | ("Randomized Controlled Trial"[MeSH Terms]) OR ("randomized controlled trial as Topic"[MeSH Terms]) OR ("controlled clinical trial"[MeSH Terms]) | | | | |
| 8# | "randomized controlled trial"[Title/Abstract] OR "random allocation"[Title/Abstract] OR "allocation"[Title/Abstract] OR "haphazard"[Title/Abstract] OR "RCT randomized controlled"[Title/Abstract] OR "randomized"[Title/Abstract] OR "controlled"[Title/Abstract] OR "clinical trial"[Title/Abstract] | | | | |
| 9# | 7# OR 8# | | | | |
| 10# | 3# AND 6# AND 9# | | | | |

| Database | Search period | Search terms |
| --- | --- | --- |
| CNKI | January 1, 2000 to July 31, 2024 | SU=(Acupuncture + Acupuncture Treatment + Acupuncture Therapy + Acupuncture Method + Acupuncture Technique + Acupuncture in Traditional Chinese Medicine) AND (Ankylosing Spondylitis + 'Ankylosing Spondylitis (as)' + Ankylosing Spondylitis Efficacy) |
| CBM | January 1, 2000 to July 31, 2024 | SU=(Acupuncture + Acupuncture Treatment + Acupuncture Therapy + Acupuncture Method + Acupuncture Technique + Acupuncture in Traditional Chinese Medicine) AND (Ankylosing Spondylitis + 'Ankylosing Spondylitis (as)' + Ankylosing Spondylitis Efficacy) |
| Wanfang | January 1, 2000 to July 31, 2024 | SU=(Acupuncture OR Acupuncture Therapy OR Acupuncture Method OR Traditional Chinese Medicine Acupuncture OR Acupuncture Treatment) AND SU=(Ankylosing Spondylitis OR Ankylosing Spondylitis Efficacy OR Ankylosing Spondylitis Treatment OR Spondyloarthritis) |
| VIP | January 1, 2000 to July 31, 2024 | U=(Acupuncture Therapy OR Acupuncture OR Acupuncture Method OR Traditional Chinese Medicine Acupuncture OR Acupuncture Treatment) AND U= (Ankylosing Spondylitis OR Spondyloarthritis OR Ankylosing Spondylitis Efficacy OR Ankylosing Spondylitis Treatment) |
| Embase | January 1, 2000 to July 31, 2024 | ('ankylosing spondylitis'/exp OR 'spondylitis ankylosing therapy'/exp OR 'spondylitis ankylosing therapy') AND ('acupuncture'/exp OR 'acupuncture' OR 'acupuncture treatment') AND [randomized controlled trial]/lim AND [english]/lim AND [embase]/lim AND [01-01-2000]/sd NOT [01-08-2024]/sd |
| Cochrane Library | January 1, 2000 to July 31, 2024 | (ankylosing spondylitis OR spondylitis ankylosing therapy OR spondylitis ankylosing therapy) AND (acupuncture OR acupuncture method OR acupuncture treatment) |
